# Complementary and alternative medicine: A narrative review of nutritional approaches for cancer-related fatigue

**DOI:** 10.1097/MD.0000000000037480

**Published:** 2024-03-15

**Authors:** Meng Li, Yue Zhang, Jimin Liu, Dong Zhang

**Affiliations:** a College of Traditional Chinese Medicine, Changchun University of Chinese Medicine, Changchun, China; b Department of Integrated Chinese and Western Medicine, Jilin Cancer Hospital, Changchun, China; c The Third Clinical Hospital of Changchun University of Traditional Chinese Medicine, Changchun, China; d College of Basic Medicine, Changchun University of Chinese Medicine, Changchun, China.

**Keywords:** cancer-related fatigue, clinical trials, complementary and alternative medicine, fatigue, nutritional approaches

## Abstract

Cancer-related fatigue (CRF) is a common symptom among patients with cancer, with a prevalence of >49%. CRF significantly affects the quality of life of patients and may also affect their overall survival. Pharmacological interventions serve as a last resort after carefully weighing the risks and benefits, with limited benefits for patients, many side effects, and adverse reactions. Compared to traditional medicine, nutritional approaches have fewer side effects, are highly accepted by patients, and do not affect the antitumor treatment of patients. Many studies have shown that nutritional approaches, as a form of complementary and alternative medicine, help improve the symptoms of CRF and the quality of life of patients. This study was designed to examine nutritional approaches to CRF and assess their effectiveness of nutritional approaches in improving CRF. We present an overview of clinical trials investigating nutritional approaches for CRF that have been published over the last 2 decades. A total of 33 records were obtained from 3 databases: Web of Science, MEDLINE, and PubMed. Some nutritional approaches, such as melatonin, PG2, and S-adenosyl-l-methionine, are potential options for CRF treatment. However, the trials included in the review varied widely in quality, most were weak in methodology, and there is currently insufficient evidence to conclude with certainty the effectiveness of nutritional approaches in reducing CRF. Therefore, the design and methods used in future complementary and alternative medicine trials should be more rigorous.

## 1. Introduction

Fatigue is a prevalent and distressing symptom that significantly affects the quality of life of patients with cancer at all stages of treatment and illness.^[1–3]^ Cancer-related fatigue (CRF) is a common condition with high prevalence. A review of 129 studies encompassing 71,568 patients revealed that nearly half (49%) of the patients experienced fatigue.^[4]^ CRF is different from other forms of fatigue because of its extreme severity and prolonged nature, along with its inability to provide relief through rest or slumber.^[5]^ During active cancer treatment, most individuals who survive cancer experience fatigue. This fatigue tends to reach its peak toward the end of the cancer treatment period and gradually decreases afterward.^[6–8]^ However, even after the completion of active treatment, a considerable number of survivors disease-free continue to experience fatigue for several years.^[9,10]^

With improvements in cancer incidence and survival rates, the use of complementary and alternative medicine (CAM) has received increasing attention.^[11]^ A growing body of research has explored the effects of CAM on CRF. CAM differs from conventional medicine. “Complementary” means together with traditional medicine, “Alternative” means in place of traditional medicine. According to the National Center for Complementary and Integrative Health,^[12]^ Complementary Health Approaches are classified according to treatment modality into nutritional, psychological, physical, and combination approaches. Nutritional approaches are similar to those of natural products such as previously defined and include dietary supplements, essential nutrients, vitamins, minerals, medicinal plants, phytochemicals, dietary plants, herbs, and spices. The 2024 National Comprehensive Cancer Network Guidelines for CRF recommend nonpharmacological interventions, including physical activity, psychosocial interventions, and cognitive behavioral therapy, as category 1 evidence.^[13]^ However, the guidelines do not recommend nutritional approaches because of the limited evidence on the effectiveness of available nutritional approaches. This narrative review aimed to assess all clinical trials on CAM, focusing on nutritional approaches. This study aimed to provide a comprehensive understanding of the benefits and risks of various nutritional approaches.

## 2. Methods

This narrative review provides an overview of various nutritional approaches to CRF that have been studied from January 2000 to December 2023. Searches were performed in 3 databases (Web of Science, MEDLINE, and PubMed) using the following terms: MeSH terms and free text: “cancer” (neoplas*, tumor*, malignan*), fatigue (lassitude, tiredness, exertion, exhaust*), and “cancer-related fatigue.” These terms were also combined with implementation study terms (e.g., “nutritional approaches,” “complementary and alternative medicine,” “dietary supplements,” “complementary medicine,” etc) using Boolean logic operators (OR, AND). The reference lists of relevant articles were also manually searched for eligible studies that met the inclusion criteria. The inclusion criteria were as follows: full-text clinical trials published in English involving participants older than 18 years in any phase of cancer treatment. The primary intervention in the trials had to be nutritional approaches and the outcome measure had to be fatigue. Review articles, case reports, trial protocols, and meta-analyses were also excluded. The reference lists of relevant articles were manually searched for eligible studies. The author conducted a comprehensive review of relevant articles, mainly the titles and abstracts. Data from the available articles are compiled in table. This table was created to facilitate documentation of the content of each trial, such as trial methods, characteristics of the population enrolled, type and stage of the tumor, degree of fatigue before intervention, duration and dose of natural products, treatment status at baseline, comments, and adverse events.

## 3. Results

The selection criteria were met and 33 records were obtained from the 3 databases. Based on the selected clinical studies, pharmacological interventions were classified as follows: I. Melatonin; II. L-carnitine; III. Ginseng; IV. Co-Enzyme Q10; V. Adenosine 5′-Triphosphate; VI. S-adenosyl-l-methionine; VII. Fatty acids; IX: PG2 injection; X: Mistletoe extracts; XI: Ginger; XII: Guarana. See Table S1, Supplemental Digital Content, http://links.lww.com/MD/L905, which illustrates the clinical trials of nutritional approaches for CRF treatment.

## 4. Discussion

### 4.1. Melatonin

Melatonin (MLT) has been used as a dietary supplement to treat various sleep disorders caused by circadian rhythm disturbances such as delayed sleep phase syndrome, shift sleep disorder, and irregular sleep-wake disorder. The mechanism by which MLT induces sleep is not through direct hypnotic effects but by resetting the body’s internal clock to match the environmental clock, resulting in normalization of both physiological and behavioral sleep patterns in patients.^[14]^ Circadian rhythm dysregulation is a central mechanism of CRF.^[15]^ Sleep patterns in patients with cancer are often characterized as abnormal and frequently disrupted, potentially resulting in skewed or unsettled circadian rhythms. Studies^[14,16]^ have shown that MLT levels are generally lower in cancer patients than in healthy controls. Therefore, MLT could theoretically alleviate CRF caused by circadian rhythm disruptions. Four clinical trials that investigated the use of MLT for CRF have been reported in the literature.

In a 1-week crossover study with randomization and placebo control, individuals at an advanced stage of cancer receiving palliative care were administered a daily dose of 20 mg MLT or placebo. This study found no significant improvement in fatigue as a result of MLT treatment.^[17]^ It is possible that the impact of MLT is overshadowed by the overall negative effects of terminal illnesses. There is a possibility that interventions that do not show any effect in the advanced stages of a disease may have a significant impact if implemented at an earlier stage.

The results of MLT intervention on fatigue symptoms in early stage breast cancer are mixed. In a randomized controlled trial (RCT) investigating the effects of MLT in patients with early stage breast cancer receiving radiotherapy and chemotherapy within 4 weeks of surgery,^[18]^ patients with severe fatigue improved significantly after MLT 18 mg daily compared to the placebo group (*P* < .001). Subsequently, an additional RCT was conducted.^[19]^ A total of 208 patients with early stage breast cancer were randomized to receive placebo or MLT (18 mg/day) from 1-week before adjuvant treatment until 2 years after the completion of adjuvant treatments. The results showed that the average fatigue score in the MLT group was significantly lower (*P* ≤ .05). In women, the use of MLT over an extended period, even after adjuvant interventions, has been demonstrated to reduce fatigue levels related to breast cancer and its treatment. Nausea is a frequently reported adverse effect of MLT. Severe nausea was observed only in the MLT group, leading to withdrawal from treatment. In contrast, a double-blind RCT study^[20]^ of 79 patients with early stage breast cancer receiving placebo or MLT (20 mg/day) from the day before radiotherapy to 2 weeks after radiotherapy showed no statistically significant differences between the arms.

In conclusion, there is a lack of significant improvements in fatigue symptoms with MLT and a limited number of studies. Sleep disorders may increase the likelihood of CRF development. Interestingly, patients with cancer may experience persistent fatigue even when they have received adequate sleep, implying that other factors contribute to the persistence of fatigue.

### 4.2. Levocarnitine

Levocarnitine (L-carnitine) plays a crucial role in energy metabolism by facilitating the oxidation of fatty acids, promoting the aerobic metabolism of carbohydrates, and aiding in the excretion of specific organic acids. Carnitine is available in both prescription and nonprescription forms. L-carnitine plays a crucial role in transporting long-chain fatty acids from the cytosol to mitochondria. This transportation process enables the oxidation of fatty acids, leading to the production of cellular energy. Muscle and cardiac cells rely on long-chain fatty acids as their primary energy source because of the substantial generation of adenosine triphosphate (ATP). Inadequate availability of carnitine might impede the efficient utilization of long-chain fatty acids in energy metabolism, which can ultimately lead to chronic fatigue. The potential contribution of carnitine deficiency to chronic fatigue is the efficient utilization of long-chain fatty acids in energy metabolism.^[21,22]^

We identified 8 studies on L-carnitine in the treatment of CRF. In all trials, L-carnitine was administered for no more than 8 weeks, with a maximum dose of 6 g/day. Five prospective open-label L-carnitine trials reported significant improvements in fatigue.

In an RCT study,^[23]^ patients with advanced cancer received L-carnitine or a placebo. The study was divided into double-blind and open-label phases, in which the results showed significantly improved fatigue on the FACT-An fatigue subscale (*P* < .03), but there was no difference between the arms. The authors then conducted another double-blind RCT, the largest clinical trial to date, to investigate the effects of L-carnitine in patients with CRF.^[24]^ A total of 209 patients with mixed cancer types receiving L-carnitine or placebo for 4 weeks showed improvement in both arms, with no statistically significant differences between the arms, suggesting a large placebo effect.

In an RCT consisting of 40 patients diagnosed with head and neck squamous cell carcinoma who underwent cisplatin-based chemoradiotherapy, notable variations were evident in the outcomes when comparing the effects of L-carnitine (administered at a dose of 1000 mg/day for a span of 8 weeks) with those of the placebo group.^[25]^ The results showed that L-carnitine helped improve health-related quality of life in patients with head and neck squamous cell carcinoma, including fatigue symptoms. In addition, 2 weeks after chemotherapy, a significant decrease in plasma total carnitine concentration was found in the control group but not in the L-carnitine group, indicating that chemotherapy impaired carnitine homeostasis. No adverse effects of the study drugs were observed.

### 4.3. Ginseng

Ginseng is an herbaceous perennial plant that is commonly used as a dietary supplement for nutritional supplementation, including different varieties of Asian ginseng (*Panax ginseng*), American ginseng (*Panax quinquefolius*), and Siberian ginseng. Siberian ginseng was not discussed in this review because it contains more active ingredients than Asian or American ginseng. Asian and American ginseng contain various ginsenosides; however, the ratios of these compounds differ between the two species. In addition to ginsenosides, ginseng also contains other components, such as polysaccharides and peptides, which can potentially exert various effects on specific tissues. In vitro data from ginseng have shown anti-inflammatory and cortisol-modulatory effects, which are consistent with the currently established hypothesis regarding the pathogenesis of CRF.^[26–28]^ The modulation of important neurotransmitters, including dopamine, noradrenaline, serotonin, and gamma-aminobutyric acid, could potentially explain how ginseng alleviates CRF.^[29–31]^

We identified 7 studies on the use of ginseng for the treatment of CRF. Four clinical trials investigated American ginseng and 3 clinical trials investigated Asian ginseng.

In a double-blind, dose-finding trial, 750, 1000, and 2000 mg/day of American ginseng was used to observe the effect of improving fatigue symptoms. No significant trend in efficacy was found for 1000 and 2000 mg/day.^[32]^ Subsequently, the authors conducted a large, double-blind, placebo-controlled RCT involving 300 patients with all types of cancers except brain or CNS lymphoma. They used the multidimensional fatigue symptom inventory-short form to assess improvements in fatigue. The results showed that a daily dosage of 2000 mg of American ginseng was significantly better than the placebo at week 8 (*P* = .003).^[33]^ In a placebo-controlled RCT study conducted on patients with primary head and neck tumors, the results indicated that the 1000 mg/day ginseng group exhibited greater improvement in interference with general activity than the control group.^[34]^

In an open-label study of 30 patients treated with high-dose Asian ginseng (800 mg daily) for 29 days, the FACIT-F scores improved in 21 patients.^[35]^ Subsequently, the authors conducted an RCT of 112 patients with advanced cancer receiving Asian ginseng (800 mg daily) or a placebo.^[36]^ The findings revealed advancements in fatigue across both the intervention and placebo cohorts; however, no intergroup differences were observed. The mechanisms of CRF are likely to differ between advanced and early stage cancers. This is because advanced cancer is linked to tumor byproducts, a heightened inflammatory response, increased brain susceptibility to the toxic effects of cancer treatment, a greater occurrence of cancer cachexia, and various other symptoms related to cancer. Finally, whether different concentrations of ginsenosides have different anti-fatigue effects remains to be investigated. In addition, in a multicenter RCT, 409 patients with colorectal cancer receiving chemotherapy were administered Korean red ginseng (2000 mg/day) or a placebo for 16 weeks. The intervention group showed an improvement in fatigue compared to the placebo group (*P* = .019).^[37]^ All adverse events were tolerable and manageable. Two RCTs conducted on Asian ginseng yielded different results. This disparity may be attributed to the fact that the study on Korean red ginseng had a larger sample size, a higher dosage of medicine, and a longer duration of treatment.

Further studies are needed to evaluate whether there are differences in the anti-fatigue effects between Asian and American ginseng. Future research should incorporate a more extensive sample size and prolong the duration of trials to effectively evaluate the genuine impact of treatment and reduce the influence of the placebo effect. In addition, based on available clinical studies, there is a lack of studies on the differences in side effects between Asian and American ginseng in the treatment of fatigue.

### 4.4. Co-Enzyme Q10

Co-Enzyme Q10 (CoQ10) naturally occurs in human organs, particularly in the heart, liver, and kidney. It is commonly used as a dietary supplement for maintaining general health. CoQ10 is a fat-soluble antioxidant that plays a crucial role in ATP production via oxidative phosphorylation. Furthermore, CoQ10’s antioxidant properties can help improve mitochondrial dysfunction. Mitochondrial dysfunction is associated with diseases related to energy metabolism. Reduced energy production and ATP depletion can result in physical disability, leading to conditions such as chronic fatigue syndrome and CRF.^[38]^ Endogenous CoQ10 deficiency has been observed in patients with cancer. CoQ10 is commonly used as a supplement in metastatic breast cancer and has been claimed to help improve cancer- and treatment-related fatigue.^[39,40]^

We identified 2 studies on the use of CoQ10 in the treatment of CRF. An RCT of patients with breast cancer (n = 57) showed a significant effect of the amino acid jelly Inner Power (IP) compared with patients receiving regular care (*P* = .005).^[41]^ IP is a semisolid, orally administered supplement containing amino acids, L-carnitine, and CoQ10. The positive therapeutic effects of IP on CRF may be attributed to components other than CoQ10. Further research is necessary to identify the specific components that contribute to the improvement in fatigue symptoms. There is insufficient evidence regarding the use of CoQ10 in CRF treatment.

### 4.5. Adenosine 5′-triphosphate

ATP is the primary source of energy in the body. It is a phosphoric acid energy substance that directly supplies energy to muscle cells. The mechanical force for muscle contraction is initiated by ATP, which triggers morphological changes in the proteins. ATP is crucial for maintaining endurance during exercise and muscle strength recovery. Inadequate ATP levels lead to fatigue due to a lack of energy. An RCT involving 57 patients with advanced cancer showed no significant difference in CRF improvement when ATP was administered intravenously for 8 weeks compared with receiving no ATP.^[42]^

### 4.6. S-adenosyl-l-methionine

S-adenosyl-l-methionine (SAM-e) can serve as a nutritional supplement to enhance joint and muscle health, ameliorate emotional states such as depression, and exhibit anti-inflammatory and analgesic effects. Moreover, SAM-e exhibits activity in the central nervous system and in liver tissues. It can be used as adjuvant therapy for cirrhosis and AIDS-associated myelopathy. Inflammation dysfunction is one of the hypotheses underlying CRF pathogenesis. Therefore, SAM-e may help improve fatigue symptoms. In an open-label pilot study, S-adenosylmethionine administration significantly improved CRF in 145 patients with colorectal cancer (n = 145) after oxaliplatin-based chemotherapy.^[43]^ Further large-scale, multicenter, RCTs are required to explore the efficacy of SAM-e as a potential treatment option for fatigue.

### 4.7. Fatty acids

Research has indicated a possible link between cancer-associated exhaustion and malnutrition, specifically, inadequate protein consumption and diminished plasma glutamine levels.^[44,45]^ Nonetheless, there remains insufficient information regarding the specific nutrients that yield benefits and the individuals that stand to gain from these interventions. Current evidence regarding the efficacy of omega-3 fatty acid supplementation in mitigating CRF is lacking. The factors responsible for these differential effects are unclear. Three clinical studies examined the effect of dietary fat on fatigue in patients with breast cancer. A pilot RCT demonstrated that foods rich in omega-3 fatty acids, along with other ingredients, reduced CRF compared to the general health education control arm.^[46]^ A multicenter, 3-arm RCT aimed to compare the effects of 3 different supplementation regimens in breast cancer survivors. The 3 regimens included 3 g fish oil + 3 g soybean oil, 6 g soybean oil, or 6 g fish oil per day for 6 weeks. Fatigue improved in all 3 groups of participants; however, the improvement was greater with soybean oil than with fish oil.^[47]^ Subsequently, the team conducted a secondary analysis of this multisite RCT^[48]^ and found that improved fatigue after fish oil supplementation was often accompanied by an increase in serum omega-3 fatty acids. Therefore, omega-3 fatty acids may be associated with an improvement in fatigue in fish supplemented with fish oil. Good nutritional status may enhance the ability to improve fatigue. Additionally, obesity and sarcopenia can lead to fatigue, and future research should explore the relationship between these types of malnutrition and fatigue.^[49,50]^

### 4.8. PG2

The PG2 injection is composed of a combination of polysaccharides extracted from the roots of *Astragalus membranaceus*. The injection of PG2, suggested to possess properties that are anti-inflammatory, immune-regulating, and beneficial for hematopoiesis, has been reported to have a notable positive impact on both fatigue and quality of life.^[51,52]^ It has been shown that PG2 helps to improve the neutrophil-to-lymphocyte ratio in lung cancer patients treated with immunotherapy.^[53]^ All 3 RCTs examined patients with advanced cancer.^[54–56]^ Additional research is necessary to broaden the utilization of PG2 injections in individuals diagnosed with early stage cancer and to substantiate the influence of these injections within this specific patient cohort. Further investigations are imperative to elucidate the mechanism by which *Astragalus polysaccharide* alleviates cancer-associated fatigue.

### 4.9. Mistletoe extracts

Mistletoe extracts are commonly used in complementary cancer therapy as immunomodulatory agents and biological response modifiers.^[57,58]^ In a multicenter, RCT with 107 patients diagnosed with locally advanced or metastatic pancreatic cancer, treatment with mistletoe extract demonstrated improvement in fatigue compared with placebo.^[59]^

### 4.10. Ginger

Ginger, the underground stem of the perennial plant, *Zingiber officinale* Roscoe, has antiemetic, anti-inflammatory, analgesic, and cardiotonic properties. In a double-blind RCT, patients with cancer were administered standardized ginger extract (1.2 g, 4 times per day) or placebo. After 3 cycles of chemotherapy, the intervention group showed significantly improved fatigue (*P* = .013) compared with the placebo group.^[60]^ Furthermore, ginger has shown potential in improving the quality of life of individuals experiencing chemotherapy-induced nausea. The addition of ginger to the diet was well tolerated, as no notable increase in unfavorable incidents was observed and only a limited number of side effects were observed.

### 4.11. Guarana

Guarana, derived from a plant indigenous to the Amazon basin, comprises an assortment of potent components such as theobromine, caffeine, saponins, and tannins. In a randomized controlled crossover study design, 75 breast cancer patients receiving active cytotoxic therapy were orally administered guarana extract at a dose of 50 mg twice daily. Compared with placebo, the treatment arm exhibited a considerable enhancement in CRF, as assessed using the FACIT-F scale (*P* < .01).^[61]^ Another RCT involving patients with solid tumors receiving guarana or placebo for 21 days found that guarana improved fatigue; however, no significant difference was observed between the placebo and intervention groups.^[62]^ Previous research investigating the effectiveness of guarana in alleviating CRF has been limited by the small number of participants and the outcomes have displayed incongruity.

## 5. Conclusion

Clinical studies on nutritional approaches to CRF have yielded mixed results. Overall, while there is evidence indicating the effectiveness of CAM in combatting CRF, there are an insufficient number of high-quality trials to completely endorse the inclusion of CAM in the standard treatment regimen for CRF. However, recent clinical trials have demonstrated the positive effects of some potential agents, such as MLT, PG2, and SAM-e, in the treatment of CRF. In addition, alpha-lipoco acid and St. John Wort have potential anti-fatigue effects, but their intervention effects on CRF have not been studied and are not discussed in this review.

Future research should undertake extensive clinical trials to evaluate the comparative advantages and adverse reactions of prospective dietary techniques. Moreover, it is imperative that these trials include a placebo wash-in phase to mitigate placebo effects. Further research on the biological mechanisms affecting fatigue is needed to provide a rationale for existing nutritional approaches and propose new approaches.

## Author contributions

**Conceptualization:** Jimin Liu.

**Project administration:** Dong Zhang.

**Supervision:** Yue Zhang, Dong Zhang.

**Writing – original draft:** Meng Li.

**Writing – review & editing:** Meng Li.

## Supplementary Material


