# Supplementary material for: Complementary and alternative medicine: A narrative review of nutritional approaches for cancer-related fatigue
Source: Medicine (Baltimore). 2024 Mar 15;103(11):e37480. doi: 10.1097/MD.0000000000037480 (PMC10939540; doi:10.1097/MD.0000000000037480)
Supplement: Supplementary file 1 [file medi-103-e37480-s001.docx]

**Table S.** Clinical trials of nutritional approaches for cancer-related fatigue

| Study | Year nation | Study design | Cancer diagnosis | Tumor staging | Intervention | Comparative Intervention | population | Key results | Adverse events |
| --- | --- | --- | --- | --- | --- | --- | --- | --- | --- |
| Melatonin | | | | | | | | | |
| C. Lund Rasmussen, M. Klee Olsen, A. Thit Johnsen, M. A. Petersen, H. Lindholm, L. Andersen, et al. | 2015 Denmark | Part 1 was a prospective, double-blind, randomized, crossover trial Part 2 was a prospective, nonrandomized, open-label study | >1 Type | IV cancer (TNM Classification) | Arm1: 1 week of melatonin at a dose of 20 mg receiving the opposite treatment for 1 week | Arm2: 1 week of placebo receiving melatonin at a dose of 20 mg for 1 week | Part 1  ITT = 72, PP = 44 Arm1: Mean age = 65, Men = 29% Arm2: Mean age = 62, Men =34% Part 2  ITT = 36, PP =18 Median age = NR, Men = NR | Part1: Analysis of the primary outcome indicated no significant difference between MLT and placebo, favoring placebo (P = 0.47) Part2: indicated an overall deterioration in the patients’ health. Fatigue scores increased by 9.7 after 3 weeks. | There was no difference in the number of acute hospitalizations or deaths occurring during the 2 weeks among the 71 patients who had consumed at least 1 capsule. |
| A. Sedighi Pashaki, K. Mohammadian, S. Afshar, et al. | 2021 Iran | Randomized, Controlled, Parallel-Group, Trial | breast cancer | stage I to III | melatonin (18mg/day) from 1 week before until 1month after the adjuvant radiotherapy | placebo from 1 week before until 1month after the adjuvant radiotherapy | ITT = 78, PP = 74 intervention: Mean age = 50.47 ± 10.79, Men = 0% placebo: Mean age = 46.05 ± 10.55, Men = 0% | After the intervention, it was significantly lower in the melatonin group (P < 0.001). | In the intervention group, 3 patients were excluded due to severe nausea and vomiting. |
| A. Sedighi Pashaki, F. Sheida, L. Moaddab Shoar, T. Hashem, et al. | 2023 Iran | randomized, controlled, parallel-group trial | breast cancer | stage I to III | melatonin (18 mg/day) from 1 week before the adjuvant treatments until 2 years after their completion | placebo from 1 week before the adjuvant treatments until 2 years after their completion | ITT = 208, PP = 183 intervention：  Median age = NR, Men = 0% placebo:  Median age = NR, Men = 0% | After the intervention, not only the mean fatigue score was significantly lower in melatonin group (P ≤ 0.05), but also a greater reduction in fatigue score in intervention group was evident (P ≤ 0.001). | Regarding the toxicity of treatments, all cases of severe nausea were observed in the melatonin group, resulting in withdrawal from the treatment |
| N. D. Mukhopadhyay, A. Khorasanchi, S. Pandey, et al. | 2023  USA | randomized double-blind placebo-controlled phase III | breast cancer | stage I to III | melatonin (20 mg/day)  starting the night before RT initiation until 2 weeks post-RT | Placebo starting the night before RT initiation until 2 weeks post-RT | ITT = 79, PP = 78 intervention：  Median age = 59.5, Men = 0% placebo:  Median age = 60.0, Men = 0% | The treatment × time for FACIT-Fatigue did not demonstrate statistical significance (P-value=0.83) in the melatonin group compared to placebo. | Melatonin was well-tolerated with no grade 3 or 4 adverse events reported. The most common side effects were headache, somnolence, and abdominal pain. |
| L-carnitine | | | | | | | | | |
| F. Graziano, R. Bisonni, V. Catalano, R. Silva, et al. | 2002 Italy | prospective open study | solid tumors | stage IV | 2g of Levocarnitine every 12h for 7 days | ** | ITT = NR, PP = 50； Median age = 51; Men = 60%` | Fatigue ameliorated in 45 patients and the mean Functional Assessment of Cancer Therapy-Fatigue score was 34.9 (P < 0.001). | NR |
| R. A. Cruciani, E. Dvorkin, P. Homel, B. Culliney, S. Malamud, L. Shaiova, et al. | 2004 USA | open-label, dose-finding study | >1 Type | all stages | The L-carnitine beginning dose was 250 mg/day, with doses increased in increments of 500 mg to a maximum target dose of 3000 mg/day. | ** | ITT = 15, PP = 13 Mean age = 55 Men = 53.8% | median BFI score at baseline was 73 versus 50 after 1 week (P = 0.009). | No adverse events related to L-carnitine were reported for any patient. |
| R. A. Cruciani, E. Dvorkin, P. Homel, S. Malamud, et al. | 2006 USA | Phase I/II open-label trial | >1 Type | advanced cancer | L-carnitine 250, 750, 1250, 1750, 2250, 2750, 3000mg/day) administered in two daily doses for 7 days. | ** | ITT = 27, PP = 21； Median age = 59.7 ± 14; Men = 63% | BFI decreased significantly, from 66±12 to 39.7 ± 26 (P < 0.001) | No patient experienced significant side effects and no toxicities were noted. |
| G. Gramignano, M. R. Lusso, et al | 2006 Italy | open-label, non-randomized study | solid tumors | advanced tumors | L-carnitine 6 g/d for 4 weeks | ** | ITT = 12, PP = 24 Median age = 60 Men = 20% | Fatigue, as measured by the Multidimensional Fatigue Symptom Inventory—Short Form, decreased significantly. | No adverse events possibly related to LC were reported for any patient. |
| R. A. Cruciani, E. Dvorkin, P. Homel, B. Culliney, S. Malamud, et al. | 2009 USA | randomized, double-blind, placebo-controlled, parallel group study | >1 Type | advanced cancer | L-carnitine (0.5 g/day for two days, followed by 1g/ day for two days, and then 2g/day for 10 days) | placebo | blinded phase: TT = 33, PP = 16; open phase: ITT = 29, PP = 27; intervention: Mean age = 66.5; Men = 53%; placebo: Mean age = 70.3, Men = 33% | Outcome data from both the double-blind and open-label phases demonstrated significantly improved fatigue on the FACT-An fatigue subscale (P < 0.03). | Only two events were determined to be related to the study drug (one constipation and one diarrhea). |
| R. A. Cruciani, J. J. Zhang, J. Manola, D. Cella, B. Ansari, et al. | 2012 USA | Phase III, Randomized, Double-Blind, Placebo-Controlled Trial | >1 Type | all stages | 2 g/d of L-carnitine for 4 weeks | placebo for 4 weeks | ITT = 376, PP = 209 intervention：  Mean age = NR, Men = 41.8% placebo:  Mean age = NR, Men = 42.3% | fatigue, measured using the BFI, improved in both arms compared with baseline. There were no statistically significant differences between arms (P = 0.57). | There were few high- grade, treatment-related toxicities. |
| K. Endo, T. Ueno, K. Ishikawa, Y. Nakanishi, S. Kondo, et al. | 2019 Japan | open label, randomized, controlled prospective study | head and neck squamous cell carcinoma | all stages | liquid L-carnitine 1000mg once daily for 8 weeks | Placebo for 8 weeks | ITT = NR, PP =40 intervention：  Mean age = 64.4, Men = 85.0% Placebo:  Median age = 65.4, Men =90.0% | After CRT, physical functioning of the control group decreased significantly (P = 0.047). However, in the L-carnitine group, this score remained unchanged (P = 0.668). | No adverse events related to L-carnitine administration were observed. |
| T. Shindo, K. Kobayashi, T. Tanaka and N. Masumori | 2019 Japan | A single-center prospective pilot study | sunitinib for unresectable or metastatic renal cell carcinoma | all stages | L-carnitine 1500 mg per day for 2weeks | ** | ITT = NR, PP = 10 Median age = 70.5; Men = NR | For six of the seven patients who had L-carnitine supplementation, the BFI score at 4 weeks decreased compared to that at 2 weeks | NR |
| Ginseng | | | | | | | | | |
| J. L. Elam, J. S. Carpenter, X. O. Shu, S. Boyapati and J. Friedmann-Gilchrist | 2006 USA | randomized, double-blind, placebo- controlled trial | Breast cancer | all stages | Group A took part in 1 week of baseline assessment, 2 weeks of placebo, followed by 4 weeks of ginseng supplement. | Group B took part in 1 week of baseline assessment, 2 weeks of ginseng, followed by 4 weeks of placebo. | ITT = 5, PP = 2 Median age = 49; Men = 0% | Because of the methodological issues encountered during the study, no results regarding the efficacy of ginseng were available. | No subjects withdrew due to undesirable side effects of the ginseng/placebo or peripheral blood draws. |
| D. L. Barton, G. S. Soori, B. A. Bauer, J. A. Sloan, P. A. Johnson, C. Figueras, et al. | 2010 USA | a randomized, double-blind, dose-finding evaluation | >1 Type | all stages | 750, 1000, or 2000 mg/day for 8 weeks | Placebo for 8 weeks | ITT = 282, PP = 175; intervention: 750mg Mean age = 70, Men = 34% intervention: 1000mg Mean age = 72, Men = 35% intervention: 2000mg Mean age = 71, Men = 32% placebo: Mean age = 69, Men = 35% | Nonsignificant trends for all outcomes were seen in favor of the 1,000- and 2,000-mg/day doses of American ginseng. | There were no significant differences in any measured toxicities between any of the arms. |
| D. L. Barton, H. Liu, S. R. Dakhil, B. Linquist, J. A. Sloan, et al. | 2013 USA | A randomized, Double-Blind trial | all cancers, other than brain or CNS lymphoma, | all stages | American ginseng 2000mg twice a day for 8 weeks | placebo for 8 weeks | ITT = 364, PP = 300; intervention:  Mean age = 55.3, Men = 19% placebo:  Mean age = 55.9, Men = 25% | A statistically significant difference was seen at 8 weeks with a change score of 20 for the ginseng group and 10.3 for the placebo group (P = 0.003). | Toxicities per self-report and CTCAE grading did not differ statistically significantly between arms |
| S. Yennurajalingam, A. Reddy, N. M. Tannir, et al. | 2015 USA | prospective, open-label study A Preliminary Report | >1 Type | all stages | 800 mg orally daily for 29 days | ** | ITT = NR, PP =30 Median age =58; Men = 50% | 21 (87%) had an improved FACIT-F score by day 15. | No severe adverse events related to the study drug were reported. |
| S. Yennurajalingam, N. M. Tannir, J. L. Williams, et al | 2017 USA | randomized, double-blind, placebo-controlled study, | >1 Type | advanced cancer | 400 mg of standardized Panax ginseng extract twice daily for 28 days | Placebo for 28 days | ITT = 127, PP = 112 intervention：Median age = 61.0, Men = 54.0% Placebo: Median age = 61.0, Men = 62.5% | Mean (SD) improvement in the FACIT-F subscale at day 29 was not significantly different in the PG than in the placebo group (P = 0.67). | There were fewer any-grade toxicities in the PG versus placebo group (P = 0.024). |
| J. W. Kim, S. W. Han, J. Y. Cho, I. J. Chung, J. G. Kim, et al. | 2020 Korea | randomized, double- blinded, placebo-controlled, parallel, multi-center trial | colorectal cancer | all stages | KRG 2000 mg/day for 16 weeks | placebo for 16 weeks | ITT = 438, PP = 409 intervention: Median age = 60.0, Men = 60.0% Placebo: Median age = 60.0, Men =61.0% | KRG led to improved CRF in the global BFI score compared with the placebo (P = 0.019) | The incidence rates of adverse events were not significantly different between groups (P = 0.937) |
| M. Guglielmo, P. Di Pede, S. Alfieri, C. Bergamini, F. Platini, et al. | 2020 Italy | A randomized, double‐blind, placebo controlled, phase II study | primary head neck tumor | medical history: at least 1 year | American ginseng 1000 mg/day for 8 weeks | Placebo for 8 weeks | ITT = NR, PP = 32 intervention：Median age = 58.0, Men = 76.0% Placebo: Median age = 55.0, Men = 60.0% | The mean of the mean values of the BFI measured at 8 weeks (end of treatment) showed a tendency to statistical significance only for the single item on interference with general activity (p = 0.06), with better performance for placebo. | There was no grade 3+ adverse events related to the study medication. |
| Coenzyme Q10 | | | | | | | | | |
| G. J. Lesser, D. Case, N. Stark, S. Williford, J. Giguere, L. A. Garino, et al. | 2013 USA | Randomized Double-Blind, Placebo-Controlled Study | breast cancer | newly diagnosed breast cancer | 300mg CoQ10 combined with 300IU vitamin E, divided into 3 daily doses for 24 weeks | placebo for 24 weeks | ITT = 236, PP = 139 intervention:  Median age = 52, Men = 0% placebo:  Median age = 50, Men = 0% | There were no significant differences between the CoQ10 and placebo arms on the POMS-F questionnaire (P = 0.257), the FACIT-F tool (P = 0.965), the FACT-B instrument (P = 0.577), or the CES- D scale (P = 0.632). | CoQ10 and placebo patients did not differ significantly in the incidence of grade 3 and 4 toxicities or any toxicity. |
| S. Iwase, T. Kawaguchi, D. Yotsumoto, T. Doi, K. Miyara, H. Odagiri, et al. | 2016 Japan | an open-label, multicenter, randomized controlled trial | Breast cancer | all stages | IP contains branched-chain amino acids (2500 mg), coenzyme Q10 (30 mg), and L-carnitine (50 mg). oral IP (125 g) once daily for 21 days | regular care consisting of recommendations for adequate exercise and relaxation | ITT = 59, PP = 57 intervention：  Median age = 49, Men = 0% Placebo:  Median age = 59, Men = 0% | The mean change in the worst level of fatigue during the past 24 h (BFI item 3) was significantly greater in the intervention group than in the control group (P = 0.005) | No severe adverse events were observed. |
| ATP | | | | | | | | | |
| S. Beijer, P. S. Hupperets, B. E. van den Borne, et al. | 2010 The Netherland | Randomized Clinical Trial | >1 Type | advanced cancer | ATP intravenously weekly (8-10 hours/week) for eight weeks | receive no ATP | ITT =100, PP =57; intervention: Mean age = 68, Men= 69% placebo: Mean age = 65, Men = 63% | Between the ATP and control groups, no statistically significant differences were observed for the large majority of outcome parameters. | NR |
| S-adenosylmethionine | | | | | | | | | |
| A. Onorato, A. Napolitano, S. Spoto, L. Incorvaia, A. Russo, D. Santini, et al | 2021 Italy | prospective open-label pilot study | colorectal cancer | all stages | S-adenosylmethionine (400 mg b.i.d.) | without AdoMet | ITT = NR, PP = 145； Median age = 63; Men = 53.1% | Mean scores from questionnaire domains like FACIT-F subscale (p = 0.006) and FACIT- F total score (p = 0.021) were higher in those receiving supplementation of AdoMet; a significant difference was maintained even after 6 months of treatment. | NR |
| Fatty acid | | | | | | | | | |
| S. M. Zick, J. Colacino, M. Cornellier, T. Khabir, K. Surnow and Z. Djuric | 2017 USA | randomized, parallel, pilot clinical trial | breast cancer | stage 0-IIIa | FRD: diet rich in fruit, vegetables, whole grains, and omega-3 fatty acid-rich foods for 3 months | GHC：improved fatigue and sleep for 3 months | ITT = 30, PP = 29 FRD: Mean age = 64.4, Men = 0% GHC: Mean age = 60.4, Men = 0% | From baseline to 3-month fatigue improved by 44 ± 39% in FRD compared to 8 ± 34% in GHC (p = 0.01); omega-3 fatty acids (p < 0.01), and ratio of omega-3: omega-6 fatty acids (p = 0.02) were significantly increased. | FRD diet could provide a non-toxic treatment strategy for persistent fatigue. |
| L. J. Peppone, J. E. Inglis, K. M. Mustian, C. E. Heckler, et al. | 2019 USA | multicenter, randomized controlled trial | Breast cancer | stage (0–III) | O3-PUFA (6 g/d) for 6 weeks O6-PUFA (6 g/d) for 6 weeks | low-dose combination of O3-/ O6-PUFA  for 6 weeks | ITT = 108, PP = 81 O3-PUFA：Mean age = 60.4 Men = 0% O6-PUFA :Mean age = 58.0 Men = 0% O3/06-PUFA Mean age = 60.7 Men = 0% | Contrary to our original hypothesis, O6-PUFA statistically significantly reduced CRF compared with O3-PUFA. | Balance of adverse events across the groups. |
| A. S. Kleckner, E. Culakova, I. R. Kleckner, E. K. Belcher, et al. | 2021 USA | a phase II multisite three-arm randomized controlled trial | Breast cancer | had had stage 0-III cancer | trial comparing 6 g fish oil, 3 g fish oil + 3 g soybean oil, or 6 g soybean oil supplementation per day for 6 weeks | trial comparing 6 g fish oil, 3 g fish oil + 3 g soybean oil, or 6 g soybean oil supplementation per day for 6 weeks | ITT = 108, PP = 85 intervention：6 g fish oil, Mean age = 58.0, Men = 0%, 3 g fish oil + 3 g soybean oil Mean age =60.7, Men = 0%, 6 g soybean oil Mean age = 60.4, Men = 0% | For those who supplemented with fish oil, greater changes in omega-3 fatty acids in the blood were associated with greater improvements in fatigue over the course of the intervention, especially greater increases in physical fatigue (p = 0.022). | NR |
| PG2 injection | | | | | | | | | |
| C. H. Wang, C. Y. Lin, J. S. Chen, C. L. Ho, K. M. Rau, J. T. Tsai, et al. | 2019 Taiwan | A Double Blind, Multi-Center, Randomized Phase IV Study | >1 Type | locally advanced, metastatic, inoperable advanced cancer | High dose arm: 500 mg for 8 weeks | Low dose arm: 250 mg for 8 weeks | ITT = 214, PP = 140 High dose arm：  Mean age = 62.2, Men = 49.55% Low dose arm:  Mean age = 62.86, Men = 64.08% | Improvement in fatigue scores by at least 10% was observed in greater than 65% of subjects after one treatment cycle compared to scores at baseline. | Drug-related adverse event rates were less than 9%. |
| W. C. Huang, K. T. Kuo, O. A. Bamodu, Y. K. Lin, C. H. Wang, et al. | 2019 Taiwan | double blind randomized controlled trial | >1 Type | advanced stage progressive metastatic cancer | high (500 mg q.d.) dose arm：PG2 three times per week per cycle of 4 weeks | low (250 mg q.d.) dose arm：PG2 three times per week per cycle of 4 weeks | ITT = 23, PP = 23 High dose arm: Mean age = 67.27 Men = 27.27% Low dose arm: Mean age = 63.67 Men = 58.33% | All 23 patients with metastatic disease treated with either low or high PG2 experienced reduced pain, nausea, vomiting, and fatigue. | NR |
| C. H. Hsieh, C. Y. Lin, C. L. Hsu, K. H. Fan, S. F. Huang, C. T. Liao, et al. | 2020 Taiwan | phase II double-blind randomized placebo-controlled trial | head and neck squamous cell carcinoma | advanced cancer | PG2 injection three times per week throughout the CCRT period | placebo throughout the CCRT period | ITT = 17, PP = 10 intervention:  Mean age = 54.2, Men = 88.9%, Placebo:  Mean age =57.7, Men = 87.5% | CRF, which was measured using the Taiwanese version of the BFI showed a moderate trend of amelioration and delay in patients receiving CCRT/PG2 compared with those receiving CCRT/placebo | The percentage of severe AE was lower in the CCRT/PG2 group than in the CRT/placebo group. |
| Mistletoe extracts | | | | | | | | | |
| W. Tröger, D. Galun, M. Reif, A. Schumann, et al. | 2014 Germany | randomized, placebo-controlled, double-blind, multicenter clinical trial | pancreatic cancer | stage III/IV | 1 ml of mistletoe extract three times per week, every week up to one year | placebo  three times per week, every week up to one year | ITT =110, PP = 107 intervention:  Mean age = NR, Men = 60.4% placebo:  Mean age = NR, Men = 51.4% | Those treated with mistletoe did better on all 6 functional scales and on 7 of 9 symptom scales, including fatigue. | It has practically no side effects, improves the quality of life, and prolongs survival. |
| Ginger | | | | | | | | | |
| W. Marx, A. L. McCarthy, K. Ried, D. McKavanagh, et al. | 2017 Australia | A Double Blind, Randomized, Placebo Controlled Trial | >1 Type | all stages | 1.2 g of a standardized ginger extract 4 times per day, for three cycles of chemotherapy. | placebo | ITT = 51, PP = 34 intervention:  Mean age = 57, Men = 34.0% Placebo:  Mean age = 59.0, Men = 41.0% | In chemotherapy Cycle 1, less fatigue (p = 0.006) than placebo participants. There were no significant results in Cycle 2. In Cycle 3, fatigue (p = 0.013) was significantly better in the intervention group compared to placebo. | Four patients experienced significant adverse events, none of which could reasonably be attributed to ginger. |
| Guarana | | | | | | | | | |
| M. P. de Oliveira Campos, R. Riechelmann, et al. | 2011 Brazil | phase II randomized, double-blind, placebo- controlled crossover trial | breast cancer | all stages | guarana 50 mg twice daily for 21 days washout 7days placebo for 21 days | placebo for 21 days washout 7 days guarana 50 mg twice daily for 21 days | ITT = 75, PP = 60; Guarana-placebo:  Mean age = 51.76, Men = 0% Placebo-guarana:  Mean age = 50.2, Men = 0% | Guarana significantly improved the FACIT-F and BFI global scores compared to placebo on days 21 and 49 (p < 0.01). | Guarana did not produce any Adverse Events grades 2, 3, or 4 toxicities. |
| A. B. del Giglio, I. Cubero Dde, T. G. Lerner, et al. | 2013 Brazil | A: uncontrolled, open study B: placebo-controlled, randomized trial | solid tumors | all stages | B: purified dry extract of guarana 37.5 mg two times per day for 21days | B: placebo for 21 days | ITT = 40, PP = 33; intervention: Mean age = 52.17, Men = 31.2% placebo: Mean age = 58, Men = 47.1% | Three weeks after randomization no significant differences in the BFI between patients randomized to guarana versus placebo. | Two instances of grade 3 toxicity in the guarana arm, which was not reported with placebo. |
| **Abbreviations:** RCT: randomized controlled trial, CRF: cancer related fatigue, ITT: Intention-To-Treat, PP: Per-Protocol, NR: not reported, RT: Radio Therapy, BFI: Brief Fatigue Inventory, SD: Standard Deviation, FACIT-F: Functional Assessment of Chronic Illness Therapy-Fatigue, CTCAE: Common Terminology Criteria for Adverse Events, LC: L-carnitine, FACT-An: Functional Assessment of Cancer Therapy-Anemia, CRT: cisplatin-based chemoradiotherapy, KRG: Korean red ginseng, FACT-B: Functional Assessment of Cancer Therapy–Breast Cancer, POMS-F: fatigue subscale of the Profile of Mood States, AdoMet: S-adenosylmethionine, FRD: fatigue reduction diet, GHC: general health curriculum, O3-PUFA: omega-3 polyunsaturated fatty acids, O6-PUFA: Omega-6 polyunsaturated fatty acids, PG2: Astragalus membranaceus, CCRT: Concurrent chemoradiotherapy. | | | | | | | | | |
